# From neuronal marker to metabolic mediator: Expanding the functional landscape of N-acetylaspartate in the central nervous system and the periphery

**DOI:** 10.4103/NRR.NRR-D-25-00931

**Published:** 2026-01-02

**Authors:** Maria Rosa Ciriolo, Fabio Ciccarone

**Affiliations:** Department of Biology, University of Rome Tor Vergata, Rome, Italy; IRCCS San Raffaele Roma, Rome, Italy

N-acetylaspartate (NAA) is a non-proteinogenic derivative of L-aspartic acid and represents one of the most abundant metabolites in the human brain, reaching concentrations of approximately 10 mM. It is predominantly synthesized in neurons via the mitochondrial enzyme N-acetyltransferase 8-like (NAT8L), which catalyzes the transfer of an acetyl group from acetyl-CoA to L-aspartate. Consequently, NAA biosynthesis is tightly linked to neuronal mitochondrial activity. First identified in the mid-20^th^ century through analyses of brain tissue extracts, NAA gained scientific interest due to its unusually high concentration in the central nervous system (CNS). Its relevance increased substantially with the advent of proton magnetic resonance spectroscopy in the 1980s, which enabled NAA non-invasive *in vivo* detection. Owing to its neuronal specificity, NAA has since become a well-established biomarker for assessing neuronal viability and function, with reduced levels reported in various neurodegenerative and neuroinflammatory disorders, including multiple sclerosis, Alzheimer’s disease, and traumatic brain injury (Moffett et al., 2007).

The distinct compartmentalization and high abundance of NAA in the CNS suggest important functional roles in neurobiology, particularly in mediating intercellular communication and metabolic cooperation between neurons and glial cells. The enzyme responsible for NAA catabolism, aspartoacylase (ASPA), which hydrolyzes NAA into acetate and aspartate, is not expressed in neurons but is predominantly localized in oligodendrocytes and, to a lesser extent, in microglia (Grønbæk-Thygesen et al., 2024). This cellular distribution underpins an NAA-based neuron-glia metabolic interaction. Although the physiological functions of NAA remain to be fully elucidated, several roles have been proposed (Moffett et al., 2007). NAA is believed to contribute to neuronal osmotic homeostasis, mitigating intracellular water fluctuations and protecting proteins from osmotic stress. However, compared to other osmolytes such as taurine, its contribution to intracerebral fluid regulation appears limited. Given its structural similarity to excitatory amino acids, NAA has been hypothesized to modulate glutamatergic neurotransmission. Although a direct role remains unconfirmed, NAA serves as a precursor for the synthesis of N-acetylaspartylglutamate (NAAG), a neuropeptide synthesized by neuron-specific NAAG synthetase. NAAG pathway reduces the free glutamate pool and helps prevent glutamate excitotoxicity, a pathological mechanism implicated in numerous neurological disorders due to glutamate synaptic accumulation. NAAG also exerts neuroprotective effects by inhibiting presynaptic glutamate release through competitive binding to metabotropic glutamate receptor 3. The catabolism of NAAG, mediated by glutamate carboxypeptidase II, predominantly localized on astrocytic membranes, leads to the release of glutamate and NAA into extracellular space. Notably, metabotropic glutamate receptor 3 is also expressed on astrocytes, where its activation enhances glutamate uptake, further limiting extracellular glutamate accumulation.

Importantly, extracellular NAA concentrations in the brain are 5- to 10-fold lower than intracellular levels, typically ranging between 100–200 µM. This pronounced gradient suggests a tightly regulated turnover involving neuronal release, glial uptake, and enzymatic activity of glutamate carboxypeptidase II. The dynamic regulation of extracellular NAA is essential for CNS homeostasis, particularly through its effects on glial cell metabolism. Oligodendrocytes, characterized by high ASPA expression, are considered the primary recipient cell type for NAA uptake and utilization. Through ASPA-mediated hydrolysis, NAA serves as a source of acetate-derived acetyl-CoA in oligodendrocytes, supporting lipid biosynthesis and energy metabolism critical for oligodendrocyte maturation and myelin formation (Dominici et al., 2023). Most evidence supporting the physiological role of NAA in myelin synthesis derives from studies on Canavan disease (CD), a fatal pediatric leukodystrophy caused by mutations in the ASPA gene (Grønbæk-Thygesen et al., 2024). In CD mouse models, ASPA deficiency leads to intramyelinic vacuolization, widespread demyelination, motor dysfunction, and early death. Restoration of ASPA expression in oligodendrocytes successfully normalizes NAA levels and reverses white matter pathology, thereby providing strong evidence for the validity of an oligodendroglia-targeted therapeutic strategy in CD (Francis et al., 2021). Interestingly, despite the absence of endogenous ASPA expression in astrocytes, ectopic overexpression of the enzyme in this cell type also ameliorates the disease phenotype, suggesting that astrocytes may, under these conditions, accelerate NAA disposal or indirectly support myelination. This indirect role may justify why astroglial targeting of ASPA was less effective in alleviating CD hallmarks than the oligodendroglial-targeted strategy (Francis et al., 2021). It is hypothesized that astrocytes may metabolize NAA to release lipid precursors or metabolic intermediates utilized by oligodendrocytes. Supporting this notion, astrocytic impairment in lipid synthesis has been linked to persistent hypomyelination, whereas similar defects in oligodendrocytes cause only transient developmental delays that resolve postnatally (Gessler et al., 2017). A striking finding is that the hypomyelination phenotype in CD can be reversed by complete deletion of NAT8L in ASPA knockout mice. Although this genetic manipulation prevents NAA accumulation and improves myelination, it does not rescue the lethality observed in CD models. In contrast, heterozygous deletion of NAT8L leads to reduced NAA levels and attenuated clinical symptoms, significantly improving survival (Maier et al., 2015). Independent studies have demonstrated that NAT8L ablation in a wild-type background as well as that disruption of the malate-aspartate shuttle, which provides mitochondrial aspartate for NAA synthesis, also causes developmental dysmyelination. Conversely, mice overexpressing NAT8L overexpression in neurons show no brain anomalies and neurological defects (von Jonquieres et al., 2018). These findings indicate that myelination is impaired at both extremes: excess NAA is toxic when ASPA is lost, whereas insufficient NAA (NAT8L or malate-aspartate shuttle loss) deprives developing glia of critical substrates for myelin lipid synthesis. Thus, NAT8L deletion is beneficial only under conditions of pathological NAA excess, and this mechanism, rather than loss of NAA-derived acetate for myelin synthesis, appears to be the principal driver of lethality in CD. The detrimental effects of NAA overaccumulation may arise from toxic extra- and intracellular actions, potentially involving receptor modulation, allosteric interference with metabolic enzymes, or dysregulation of signaling cascades inside the brain but also at the periphery.

Emerging evidence suggests that NAA modulates inflammatory processes both within the CNS and in peripheral tissues. In the brain, NAA appears to exert predominantly anti-inflammatory effects. *In vitro* studies using human astroglial cell lines have shown that NAA reduces the expression of various pro-inflammatory cytokines following activation by inflammatory stimuli. A more defined role has been demonstrated in murine microglia, where NAA supports a resting, surveillance-like metabolic phenotype by enhancing lipid turnover and mitochondrial oxidative metabolism, processes facilitated by ASPA-mediated acetate supply (Felice et al. 2024). Upon stimulation with lipopolysaccharide and interferon-γ, NAA-treated microglia display a blunted pro-inflammatory response. This effect is mediated, at least in part, by the activation of histone deacetylases, which epigenetically repress the transcription of inflammatory genes. Notably, these effects are evident at extracellular NAA concentrations equivalent to physiological brain levels or higher, whereas 10-fold lower concentrations are ineffective. Given that prolonged neuroinflammation contributes to neuronal damage, NAA may play a physiological role in dampening inflammatory responses under both baseline and pathological conditions. During neuronal injury, often accompanied by an inflammatory burst, extracellular NAA concentrations increase locally, potentially serving as a negative feedback signal to limit immune activation. Conversely, the reduced NAA levels typically observed in neurodegenerative diseases may fail to restrain glial inflammation, thereby contributing to the chronic neuroinflammatory milieu that characterizes these disorders. The inability of short-term NAA administration to suppress microglial activation suggests that its anti-inflammatory effects are not mediated by receptor-based mechanisms such as N-methyl-D-aspartate receptor (NMDAR) signaling. In contrast to its activity in the CNS, NAA has been shown to act through receptor-mediated mechanisms in peripheral immune cells. In macrophages, NAA produced by tumor cells suppresses pro-inflammatory signaling via direct inhibition of NMDAR, which is known to synergize with other inflammatory pathways (Menga et al., 2021). The discrepancy in NAA’s mechanism of action between microglia and peripheral macrophages may result from differences in NMDAR subunit composition, co-receptor expression, or extracellular ligand availability. These findings indicate that immunomodulatory functions of NAA are context-dependent and vary across tissue types. Additionally, in peripheral tissues such as the gastrointestinal tract, NAA has been observed to induce pro-inflammatory responses, as demonstrated in rat stomach tissue and suggesting a connection with the gastrointestinal defects occurring in CD.

Besides this first evidence, during the last decades several works have focused attention on the influence of NAA on peripheral tissues. Notably, high expression levels of NAT8L have been detected in brown adipose tissue (BAT), a metabolically active tissue critical for thermoregulation and energy expenditure. BAT generates heat via non-shivering thermogenesis, a process driven by mitochondrial uncoupling and reliant on robust lipid catabolism (Bogner-Strauss, 2017). In this context, NAT8L-mediated NAA synthesis facilitates the export of excess mitochondrial acetyl-CoA to the cytosol. Here, NAA is hydrolyzed by ASPA, producing acetate that serves as a precursor for de novo lipid synthesis, thereby sustaining thermogenesis-associated lipid turnover. This mechanism represents a cell-autonomous NAA recycling pathway – distinct from the canonical intercellular route involving neuronal synthesis and glial utilization – underscoring the versatility of the NAA-ASPA metabolic axis. In amyotrophic lateral sclerosis (ALS), BAT exhibits a distinct metabolic adaptation characterized by overactivation of thermogenesis. However, this is paradoxically accompanied by reduced ASPA expression in BAT and elevated NAA levels in both BAT and systemic circulation. These findings suggest that, unlike in physiological conditions, BAT in ALS may produce and release NAA for utilization by other tissues, potentially compensating for widespread metabolic stress. The observed increase in circulating NAA levels supports the hypothesis that NAA functions as a systemic energy-transfer metabolite under pathophysiological conditions, particularly when metabolic demands in peripheral tissues, such as skeletal muscle in ALS, are elevated. Consistently, recent studies have demonstrated that NAA plays a functional role in regulating skeletal muscle homeostasis. In cultured myotubes, NAA treatment induces a metabolic shift favoring lipid-driven oxidative metabolism at the expense of glycolysis. This reprogramming is dependent on ASPA-mediated lipid turnover, mirroring the mechanism observed in brown adipocytes. Morphologically, NAA-treated myotubes display reduced diameters accompanied by enhanced protein turnover, likely reflecting myofilament remodeling and organelle reorganization. Importantly, these changes confer increased resistance to various atrophic stimuli (Castelli et al., 2024). In the context of neuromuscular diseases such as ALS, future studies need to clarify whether NAA release in response to neuronal damage at the neuromuscular junction may support a compensatory adaptation in skeletal muscle to mitigate atrophy.

From its initially described roles in the CNS to emerging functions in peripheral tissues, NAA is increasingly recognized as a key signaling metabolite. This is supported by the tissue-specific expression pattern of NAT8L, which is largely confined to the brain, BAT, kidney, and breast tissue, whereas ASPA is more ubiquitously expressed. This metabolic asymmetry suggests that NAA may exert distinct functions both in its intact form and through its breakdown products. NAA inherent functions certainly occur in neurons or in the extracellular space. In neurons, high intracellular concentrations of NAA may participate in allosteric modulation of enzymatic reactions other than in osmoregulation. *In vitro* analyses have shown that NAA can inhibit antioxidant enzymes such as catalase and glutathione peroxidase, implicating it in oxidative stress pathways relevant to CD.

Although NAA is not classified as a neurotransmitter, computational models have predicted its potential to act as a competitive antagonist at NMDAR, possibly interfering with glutamate binding (Menga et al., 2021). Experimental findings, however, are inconsistent: NAA has been reported to activate NMDAR at high concentrations in neuronal cultures, exhibit no effect in glial cells, or inhibit NMDAR signaling in macrophages at low concentrations. The physiological relevance of these receptor interactions in the CNS remains to be established. What is instead becoming increasingly evident is the importance of NAA-mediated acetate supply following its intracellular hydrolysis by ASPA in recipient cells. In this context, the central role of acetyl-CoA is apparent, given that this metabolite sits at the crossroads of cellular metabolism linking catabolic and anabolic pathways as well as epigenetic regulation. Because acetyl-CoA is membrane-impermeable, its subcellular compartmentalization is crucial for orchestrating specific signaling pathways. The enzyme acyl-CoA synthetase short-chain family member 2 (ACSS2), which activates acetate to acetyl-CoA, is co-localized with ASPA in both the cytoplasm and nucleus, positioning the NAA-ASPA-ACSS2 axis as a key modulator of metabolic and epigenetic processes (Moffett et al., 2020).

**Figure 1:**
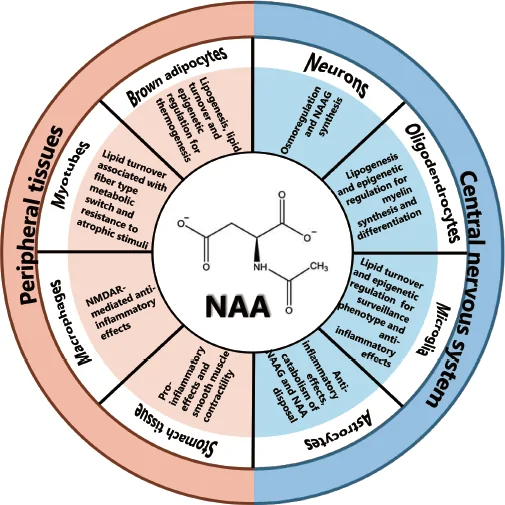
Summary of the role played by NAA in the central nervous system and the periphery. The schematic highlights how NAA may exert metabolic functions by providing acetyl coenzyme A for lipogenesis and acetylation processes in both glial cells and peripheral tissues. Notably, NAA also plays a role in the modulation of inflammatory processes, acting as a signaling molecule. NAA: N-acetylaspartate; NAAG: N-acetylaspartylglutamate; NMDAR: N-methyl-D-aspartate receptor.

While NAA-derived acetate consistently supports cytosolic lipogenesis across multiple experimental models, increased acetyl-CoA availability does not necessarily translate into heightened protein acetylation. In several systems, including brown adipocytes, oligodendrocytes, and microglia, NAA treatment results in no significant change or even a decrease in histone or global lysine acetylation. This phenomenon is often accompanied by upregulation of histone deacetylase enzymes, suggesting a compensatory epigenetic mechanism aimed at preventing hyperacetylation. Furthermore, in oligodendrocytes, NAA administration increases α-ketoglutarate levels while reducing histone H3 methylation marks. As α-ketoglutarate serves as a co-substrate for histone and DNA demethylases, but also other dioxygenases involved in extracellular matrix remodeling and cellular stress responses, these observations imply broader regulatory effects of NAA on multiple biological pathways. Collectively, these findings support the notion that NAA is a multifaceted metabolite, exerting its effects both through its intact molecular form and via its metabolic derivatives, within the CNS as well as in peripheral tissues (**Figure 1**). In some respects, NAA may exhibit functional similarities to ketone bodies, being synthesized in a specific tissue and utilized by others. This dual role, as both an energy carrier and a signaling metabolite, underscores its potential relevance in intercellular metabolic communication.

*This work was partially supported by Ricerca Scientifica d’Ateneo 2021 (E83C22000160005 to FC) University of Rome “Tor Vergata”; Italian Ministry of Health - Ricerca Corrente; #NEXTGENERATIONEU (NGEU) funded by the Ministry of University and Research (MUR), National Recovery and Resilience Plan (NRRP), project MNESYS (PE0000006 to MRC) – (DN. 1553 11.10.2022).*
